# Supplementary material for: Associations of Tinnitus Incidence with Use of Tumor Necrosis Factor-Alpha Inhibitors among Patients with Autoimmune Conditions
Source: J Clin Med. 2023 Mar 1;12(5):1935. doi: 10.3390/jcm12051935 (PMC10004033; doi:10.3390/jcm12051935)
Supplement: Supplementary file 1 [file jcm-12-01935-s001.zip › jcm-2199566-supplementary.pdf]

SUPPLEMENTAL MATERIAL *for* "Associations of tinnitus incidence with use of tumor necrosis factor-alpha inhibitors among patients with autoimmune conditions"

- Table S1. Diagnostic and treatment codes used in the database search and propensity score matching
- Figure S1. Sample selection flowchart for the comparisons of tinnitus incidence in the main sample (No-TNFα and Yes-TNFα)
- Table S2. Demographic and clinical characteristics of the TNFα-FP and TNFα-AB cohorts, before and after propensity score matching
- Table S3. Demographic and clinical characteristics of the TNFα-AB cohort and a randomly selected No-TNFα cohort, before and after propensity score matching
- Table S4. Demographic and clinical characteristics of the TNFα-FP cohort and a randomly selected No-TNFα cohort, before and after propensity score matching
- Table S5. Demographic and clinical characteristics of the Yes-TNFα cohort and a randomly selected No-TNFα cohort (age 30-50 years), before and after propensity score matching
- Table S6. Demographic and clinical characteristics of the Yes-TNFα cohort and a randomly selected No-TNFα cohort (age 51-70 years), before and after propensity score matching
- Table S7. Demographic and clinical characteristics of the Yes-TNFα cohort with ≥6 months of anti-TNFα use and a randomly selected No-TNFα cohort, before and after propensity score matching
- Table S8. Demographic and clinical characteristics of the Yes-TNFα cohort with ≥12 months of anti-TNFα use and a randomly selected No-TNFα cohort, before and after propensity score matching
- Table S9. Demographic and clinical characteristics of the Yes-TNFα cohort with RA and a randomly selected No-TNFα cohort with RA, before and after propensity score matching
- Figure S2. Unmatched, basic-matched, and propensity score-matched comparisons of tinnitus incidence between patients with autoimmune disorders, by use of TNFα-AB (a-c) or TNFα-FP (d-f) compared to no anti-TNFα therapy
- Figure S3. Unmatched, basic-matched, and propensity score-matched comparisons of tinnitus incidence between patients with autoimmune disorders, by use of anti-TNFα for ≥6 months (a-c) or ≥12 months (d-f) compared to no anti-TNFα therapy
- Figure S4. Unmatched (a), basic-matched (b), and propensity score-matched (c) comparisons of tinnitus incidence between patients with RA, by use of anti-TNFα therapy or no anti-TNFα therapy

Table S1. Diagnostic and treatment codes used database search and propensity score matching

	ICD codes	ATC codes
**Tinnitus**	ICD9: 388.3; ICD10: H93.1	
**Autoimmune disorders**		
Rheumatoid arthritis	ICD9: 714; ICD10: M06	---
Psoriasis	ICD9: 696; ICD10: L40	---
Crohn's disease	ICD9: 555-558.99; ICD10: K50	---
Ulcerative colitis	ICD9: 556; ICD10: K51	---
Noninfectious uveitis	ICD9: 364.04; ICD10: H20.04	---
Ankylosing spondylosis	ICD9: 720.0; ICD10: M45	
**Anti-TNFα therapies**		L04AB
Monoclonal antibodies		
Infliximab		L04AB02
Adalimumab		L04AB04
Certolizumab pegol		L04AB05
Golimumab		L04AB06
Fusion protein		
Etanercept		L04AB01
**CCI comorbidities**		
Malignancy	ICD9: 140.x-172.x, 174.x-195.8, 200.x-208.x, 238.6; ICD10: C00.x-C26.x, C30.x-C34.x, C37.x-C41.x, C43.x, C45.x-C58.x, C60.x-C76.x, C81.x-C85.x, C88.x, C90.x-C97.x	
Metastatic solid tumor	ICD9: 196.x-199.x; ICD10: C77.x-C80.x	
Diabetes	ICD9: 250.0-250.3, 250.8, 250.9; ICD10: E10.0, E10.l, E10.6, E10.8, E10.9, E11.0, E11.1, E11.6, E11.8, E11.9, E12.0, E12.1, E12.6, E12.8, E12.9, E13.0, E13.1, E13.6, E13.8, E13.9, E14.0, E14.1, E14.6, E14.8, E14.9	
Diabetes w/ complications	ICD9: 250.4-250.7; ICD10: E10.2-E10.5, E10.7, E11.2-E11.5, E11.7, E12.2-E12.5, E12.7, E13.2-E13.5, E13.7, E14.2-E14.5, E14.7	
Congestive heart failure	ICD9: 398.91, 402.01, 402.11, 402.91, 404.01, 404.03, 404.11, 404.13, 404.91, 404.93, 425.4-425.9, 428.x; ICD10: I09.9,I11.0, I13.0, I13.2, I25.5, I42.0, I42.5-I42.9, I43.x, I50.x, P29.0	
Myocardial infarction	ICD9: 410.x, 412.x; ICD10: I21.x, I22.x, I25.2	
Peripheral vascular disease	ICD9: 093.0, 437.3, 440.x, 441.x, 443.1-443.9, 447.1, 557.1, 557.9, V43.4; ICD10: I70.x, I71.x, I73.1, I73.8, I73.9, I77.1, I79.0, I79.2, K55.1, K55.8, K55.9, Z95.8, Z95.9	

Chronic pulmonary disease	ICD9: 416.8, 416.9, 490.x-505.x, 506.4, 508.1, 508.8; ICD10: I27.8, I27.9, J40.x-J47.x, J60.x-J67.x, J68.4, J70.1, J70.3	
Cerebrovascular disease	ICD9: 362.34, 430.x-438.x; ICD10: G45.x, G46.x, H34.0, I60.x-I69.x	
Dementia	ICD9: 290.x, 294.1, 331.2; ICD10: F00.x-F03.x, F05.1, G30.x, G31.1	
Hemiparaplegia	ICD9: 334.1, 342.x, 343.x, 344.0-344.6, 344.9; ICD10: G04.1, G11.4, G80.1, G80.2, G81.x, G82.x, G83.0-G83.4, G83.9	
Mild liver disease	ICD9: 070.22, 070.23, 070.32, 070.33, 070.44, 070.54, 070.6, 070.9, 570.x, 571.x, 573.3, 573.4, 573.8, 573.9, V42.7; ICD10: B18.x, K70.0-K70.3, K70.9, K71.3-K71.5, K71.7, K73.x, K74.x, K76.0, K76.2-K76.4, K76.8, K76.9, Z94.4	
Moderate or severe liver disease	ICD9: 456.0-456.2, 572.2-572.8; ICD10: I85.0, I85.9, I86.4, I98.2, K70.4, K71.1, K72.1, K72.9, K76.5, K76.6, K76.7	
Renal disease	ICD9: 403.01, 403.11, 403.91, 404.02, 404.03, 404.12, 404.13, 404.92, 404.93, 582.x, 583.0-583.7, 585.x, 586.x, 588.0, V42.0, V45.1, V56.x; ICD10: I12.0, I13.1, N03.2-N03.7, N05.2-N05.7, N18.x, N19.x, N25.0, Z49.0-Z49.2, Z94.0, Z99.2	
Peptic ulcer disease	ICD9: 531.x-534.x; ICD10: K25.x-K28.x	
Rheumatic disease	ICD9: 446.5, 710.0-710.4, 714.0-714.2, 714.8, 725.x; ICD10: M05.x, M06.x, M31.5, M32.x-M34.x, M35.1, M35.3, M36.0	
HIV	ICD9: 042.x-044.x; ICD10: B20.x-B22.x, B24.x	

Caption: Abbreviations: ATC, Anatomical Therapeutic Chemical; CCI, Charlson Comorbidity Index; HIV, human immunodeficiency virus; ICD9/10, International Classification of Diseases, 9th/10th editions; TNFα, tumor necrosis factor-alpha.

Figure S1. Sample selection flowchart for the comparisons of tinnitus incidence in the main sample (No-TNFα and Yes-TNFα)

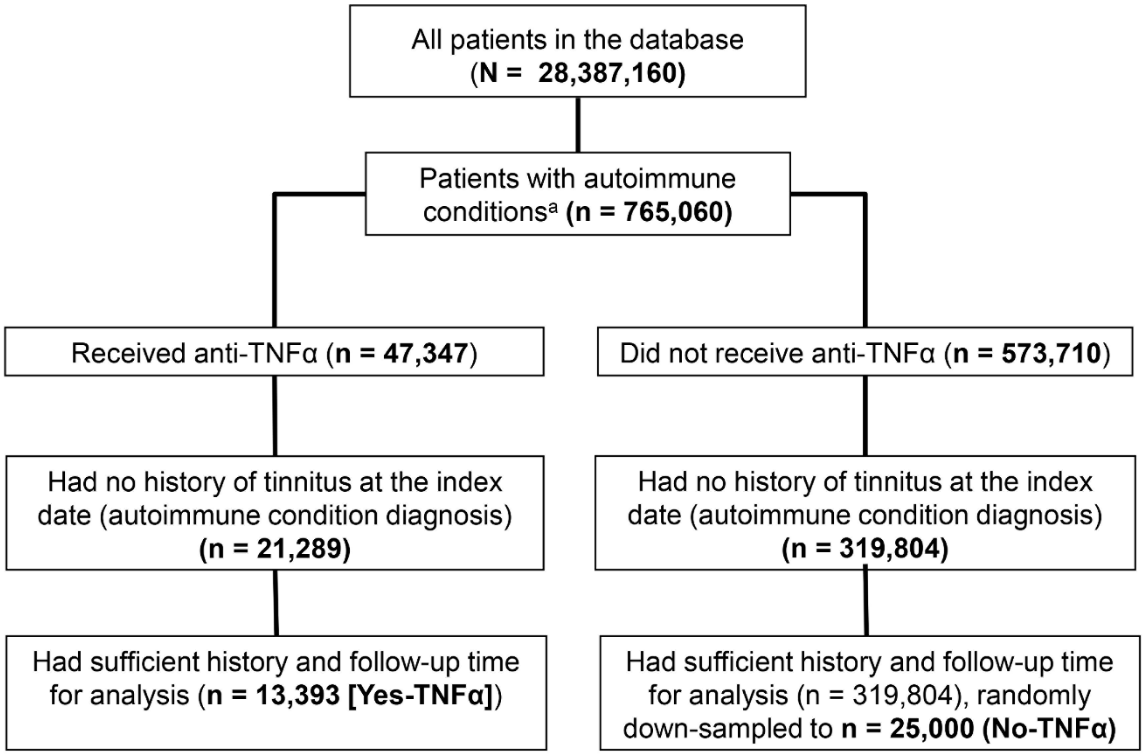

Abbreviations: TNFα, tumor necrosis factor-alpha. Note: [a] Autoimmune disorders (rheumatoid arthritis, psoriasis/psoriatic arthritis, ulcerative colitis, ankylosing spondylosis, Crohn’s disease, and non-infective uveitis) and anti-TNFα therapies were identified based on diagnosis and drug codes listed in Table S1.

Table S2. Demographic and clinical characteristics of the TNFα-FP and TNFα-AB cohorts, before and after propensity score matching

	Autoimmune disorder + TNFα cohorts[a]			
	Before matching		After matching	
	TNFα-FP N=2397	TNFα-AB N=9471	TNFα-FP N=1927	TNFα-AB N=1927
**Duration of follow-up, mean [SD] days**	1635.9 [1061.2]	1529.3 [1032]	1777.3 [1083.9]	1764.2 [1095.9]
**Sex, n (%) female**	1601 (66.8%)	6179 (65.2%)	1308 (67.9%)	1329 (69.0%)
**Age, years**				
Mean [SD]	56.5 [12.8]	52.3 [15.2]	56.0 [12.7]	56.7 [14.2]
Distribution, n (%)				
18-29	68(2.8%)	898 (9.5%)	59 (3.1%)	112 (5.8%)
30-39	199 (8.3%)	1236 (13.1%)	168 (8.7%)	165 (8.6%)
40-49	416 (17.4%)	1805 (19.1%)	347 (18.0%)	232 (12.0%)
50-59	725 (30.2%)	2331 (24.6%)	591 (30.7%)	522 (27.1%)
60-69	629 (26.2%)	2050 (21.6%)	504 (26.2%)	567 (29.4%)
70-79	300 (12.5%)	906 (10.1%)	221 (11.5%)	290 (15.0%)
80-89	60 (2.5%)	191 (2.0%)	37 (1.9%)	39 (2.0%)
**Race/ethnicity, n (%)**				
White	1594 (66.5%)	6226 (65.7%)	1215 (63.1%)	1218 (63.2%)
Black	151 (6.3%)	610 (6.4%)	138 (7.2%)	127 (6.6%)
Asian	14 (0.6%)	76 (0.8%)	10 (0.6%)	10 (0.6%)
Other [b]	638 (26.6%)	2559 (27.0%)	562 (29.2%)	571 (29.6%)
**CCI**				
Mean score [SD]	2.5 [1.7]	1.9 [1.8]	2.5 [1.7]	2.6 [1.8]
Component disorders, n (%)				
Malignancy	65 (2.7%)	167 (1.8%)	58 (3.0%)	59 (3.1%)
Metastatic solid tumor	0 (0%)	10 (0.1%)	0 (0%)	4 (0.1%)
Diabetes	313 (13.1%)	1005 (10.6%)	264 (13.7%)	293 (15.2%)
Diabetes w/ complications	58 (2.4%)	215 (2.3%)	54 (2.8%)	68 (3.5%)
Congestive heart failure	36 (1.5%)	126 (1.3%)	35 (1.8%)	37 (1.9%)
Myocardial infarction	21 (0.9%)	49 (0.5%)	19 (1.0%)	10 (0.5%)
Peripheral vascular disease	56 (2.3%)	185 (2.0%)	50 (2.6%)	58 (3.0%)
Chronic pulmonary disease	285 (11.9%)	1052 (11.0%)	245 (12.7%)	291 (15.1%)
Cerebrovascular disease	52 (2.2%)	188 (2.1%)	45 (2.3%)	61 (3.2%)
Dementia	1 (<0.1%)	12 (0.1%)	1 (0.1%)	5 (0.3%)
Hemiparaplegia	6 (0.3%)	12 (0.1%)	6 (0.3%)	9 (0.5%)
Mild liver disease	52 (2.2%)	193 (2.0%)	45 (2.3%)	48 (2.5%)
Severe liver disease	3 (0.1%)	5 (0.1%)	2(0.1%)	2 (0.1%)
Renal disease	63 (2.6%)	204 (2.2%)	55 (2.9%)	65 (3.8%)

Peptic ulcer disease	11 (0.5%)	104 (1.1%)	10 (0.5%)	31 (1.6%)
Rheumatic disease	1630 (68.0%)	3981 (42.0%)	1306 (67.8%)	1057 (54.9%)
HIV	3 (0.1%)	4 (<0.1%)	3 (0.2%)	1 (0.1%)

Caption: Grey highlight indicates characteristics with standardized mean difference >0.25 between groups. Patients in the two cohorts were propensity score-matched on sex, age, race/ethnicity, and CCI score. Abbreviations: AB, monoclonal antibody; anti-TNFα, tumor necrosis factor-alpha inhibitor; CCI, Charlson Comorbidity Index; EHR, electronic health records; FP, fusion protein; HIV, human immunodeficiency virus; SD, standard deviation. Note: [a] Demographic and clinical characteristics are among patients who met the inclusion criteria for history (90 days) and follow-up (6 months) in the EHR database. [b] 'Other' includes all other race/ethnicities as well as when this information was missing.

Table S3. Demographic and clinical characteristics of the TNFα-AB cohort and a randomly selected No-TNFα cohort, before and after propensity score matching

	Autoimmune disorders cohorts[a]			
	Before matching		After matching	
	No-TNFα N=25,000[b]	TNFα-AB N=10,859	No-TNFα N=9,247	TNFα-AB N=9,247
**Duration of follow-up, mean [SD] days**	1422.2 [993.1]	1567.6 [1042.5]	1390.2 [967.0]	1624.7 [1064.9]
**Sex, n (%) female**	16213 (64.9%)	7186 (66.2%)	6059 (65.5%)	6064 (65.6%)
**Age, years**				
Mean [SD]	56.2 [17.5]	52.2 [14.9]	53.1 [17.2]	52.8 [15.0]
Distribution, n (%)				
18-29	2534 (10.1%)	978 (9.0%)	1125 (12.2%)	830 (9.0%)
30-39	2542 (10.2%)	1360 (12.5%)	1194 (12.9%)	1133 (12.3%)
40-49	3308 (13.2%)	2097 (19.3%)	1456 (15.7%)	1701 (18.4%)
50-59	4992 (20.0%)	2764 (25.5%)	1902 (20.6%)	2368 (25.6%)
60-69	5474 (21.9%)	2356 (21.7%)	1892 (20.5%)	2048 (22.1%)
70-79	4316 (17.3%)	1092 (10.1%)	1243 (13.4%)	973 (10.5%)
80-89	1834 (7.3%)	212 (2%)	435 (4.7%)	194 (2.1%)
**Race/ethnicity, n (%)**				
White	15421 (61.7%)	7126 (65.6%)	5967 (64.5%)	5919 (64.0%)
Black	1924 (7.7%)	679 (6.3%)	611 (6.6%)	595 (6.4%)
Asian	230 (0.9%)	90 (0.8%)	76 (0.8%)	81 (0.9%)
Other[c]	7425 (29.7%)	2964 (27.3%)	2593 (28%)	2652 (28.7%)
**CCI**				
Mean score [SD]	2.6 [2.4]	2 [1.8]	2.2 [2.1]	2 [1.8]
Component disorders, n (%)				
Malignancy	1158 (4.6%)	185 (1.7%)	290 (3.1%)	170 (1.8%)
Metastatic solid tumor	67 (0.3%)	11 (0.1%)	11 (0.1%)	10 (0.1%)
Diabetes	4156 (16.6%)	1186 (10.9%)	1252 (13.5%)	1017 (11.0%)
Diabetes w/ complications	1107 (4.4%)	260 (2.4%)	307 (3.3%)	226 (2.4%)
Congestive heart failure	962 (3.9%)	146 (1.3%)	208 (2.3%)	128 (1.4%)
Myocardial infarction	271 (1.1%)	55 (0.5%)	67 (0.7%)	49 (0.5%)
Peripheral vascular disease	1427 (5.7%)	211 (1.9%)	347 (3.8%)	197 (2.1%)
Chronic pulmonary disease	4916 (19.7%)	1235 (11.4%)	1573 (17.0%)	1061 (11.5%)
Cerebrovascular disease	1240 (5.0%)	221 (2.0%)	263 (2.8%)	196 (2.1%)
Dementia	193 (0.8%)	14 (0.1%)	41 (0.4%)	11 (0.1%)
Hemiparaplegia	83 (0.3%)	12 (0.1%)	18 (0.2%)	9 (0.1%)
Mild liver disease	848 (3.4%)	237 (2.2%)	270 (2.9%)	203 (2.2%)
Severe liver disease	47 (0.2%)	7 (0.1%)	10 (0.1%)	7 (0.1%)
Renal disease	1376 (5.5%)	241 (2.2%)	336 (3.6%)	213 (2.3%)

Peptic ulcer disease	314 (1.3%)	110 (1.0%)	70 (0.8%)	105 (1.1%)
Rheumatic disease	7241 (29.0%)	4875 (44.9%)	2737 (29.6%)	4051 (43.8%)
HIV	43 (0.2%)	4 (<0.1%)	14 (0.2%)	3 (0.03%)

Caption: The TNFα-AB cohort used infliximab, adalimumab, certolizumab pegol, or golimumab. Grey highlight indicates characteristics with standardized mean difference >0.25 between groups. Patients in the two cohorts were propensity score-matched on sex, age, race/ethnicity, and CCI score. Abbreviations: AB, monoclonal antibody; anti-TNFα, tumor necrosis factor-alpha inhibitor; CCI, Charlson Comorbidity Index; EHR, electronic health records; HIV, human immunodeficiency virus; SD, standard deviation. Notes: [a] Demographic and clinical characteristics are among patients who met the inclusion criteria for history (90 days) and follow-up (6 months) in the EHR database. [b] The cohort was comprised of 25,000 randomly selected patients with no anti-TNFα use. [c] Other' includes all other race/ethnicities as well as when this information was missing.

Table S4. Demographic and clinical characteristics of the TNFα-FP cohort and a randomly selected No-TNFα cohort, before and after propensity score matching

	Autoimmune disorders cohorts[a]			
	Before matching		After matching	
	No-TNFα N=25,000[b]	TNFα-FP N=3,506	No-TNFα N=3,477	TNFα-FP N=3,477
**Duration of follow-up, mean [SD] days**	1422.5 [993.1]	1687.8 [1055.2]	1438.1 [981.6]	1689.5 [1056.8]
**Sex, n (%) female**	15981 (63.9%)	2420 (69.0%)	2383 (68.5%)	2398 (69.0%)
**Age, years**				
Mean [SD]	55.1 [17.1]	55.4 [13.0]	55.0 [17.2]	55.3 [13.0]
Distribution, n (%)				
18-29	2418 (9.7%)	135 (3.9%)	365 (10.5%)	135 (3.9%)
30-39	2571 (10.3%)	316 (9.0%)	397 (11.4%)	315 (9.1%)
40-49	3460 (13.8%)	667 (19.0%)	442 (12.7%)	662 (19.0%)
50-59	5095 (20.4%)	1068 (30.5%)	672 (20.7%)	1058 (30.4%)
60-69	5536 (22.1%)	853 (24.3%)	766 (22.0%)	845 (24.3%)
70-79	4239 (17.0%)	395 (11.3%)	534 (15.4%)	390 (11.2%)
80-89	1681 (6.7%)	72 (2.1%)	213 (6.1%)	72 (2.1%)
**Race/ethnicity, n (%)**				
White	15489 (62.0%)	2300 (65.6%)	2302 (66.2%)	2280 (65.6%)
Black	1920 (7.7%)	199 (5.7%)	221 (6.4%)	197 (5.7%)
Asian	203 (0.8%)	28 (0.8%)	32 (0.9%)	28 (0.8%)
Other[c]	7388 (29.6%)	979 (27.9%)	922 (26.5%)	972 (28.0%)
**CCI**				
Mean score [SD]	2.6 [2.4]	2.4 [1.7]	2.7 [2.4]	2.4 [1.7]
Component disorders, n (%)				
Malignancy	1187 (4.8%)	76 (2.2%)	147 (4.2%)	73 (2.1%)
Metastatic solid tumor	69 (0.3%)	1 (<0.1%)	13 (0.4%)	1 (<0.1%)
Diabetes	4131 (16.5%)	466 (13.3%)	586 (16.9%)	459 (13.2%)
Diabetes w/ complications	1114 (4.5%)	95 (2.7%)	170 (4.9%)	93 (2.7%)
Congestive heart failure	921 (3.7%)	52 (1.5%)	127 (3.7%)	52 (1.5%)
Myocardial infarction	264 (1.1%)	30 (0.9%)	29 (0.8%)	29 (0.8%)
Peripheral vascular disease	1394 (5.6%)	77 (2.2%)	191 (5.5%)	77 (2.2%)
Chronic pulmonary disease	4758 (19.0%)	427 (12.2%)	691 (19.9%)	425 (12.2%)
Cerebrovascular disease	1145 (4.9%)	80 (2.3%)	148 (4.3%)	80 (2.3%)
Dementia	178 (0.7%)	3 (0.1%)	18 (0.5%)	3 (0.1%)
Hemiparaplegia	101 (0.4%)	6 (0.2%)	10 (0.3%)	6 (0.2%)
Mild liver disease	835 (3.4%)	91 (2.6%)	149 (4.3%)	89 (2.6%)
Severe liver disease	46 (0.2%)	5 (0.1%)	11 (0.3%)	5 (0.1%)
Renal disease	1295 (5.2%)	81 (2.5%)	184 (5.3%)	90 (2.6%)

Peptic ulcer disease	346 (1.4%)	16 (0.5%)	55 (1.6%)	16 (0.5%)
Rheumatic disease	7258 (29.0%)	2366 (67.5%)	1299 (37.4%)	2341 (67.3%)
HIV	40 (0.2%)	3 (0.1%)	9 (0.3%)	3 (0.1%)

Caption: The TNFα-FP cohort used etanercept. Grey highlight indicates characteristics with standardized mean difference >0.25 between groups. Patients in the two cohorts were propensity score-matched on sex, age, race/ethnicity, and CCI score. Abbreviations: anti-TNFα, tumor necrosis factor-alpha inhibitor; CCI, Charlson Comorbidity Index; EHR, electronic health records; FP, fusion protein; HIV, human immunodeficiency virus; SD, standard deviation. Notes: [a] Demographic and clinical characteristics are among patients who met the inclusion criteria for history (90 days) and follow-up (6 months) in the EHR database. [b] The cohort was comprised of 25,000 randomly selected patients with no ant-TNFα use. [c] Other' includes all other race/ethnicities as well as when this information was missing.

Table S5. Demographic and clinical characteristics of the Yes-TNFα cohort and a randomly selected No-TNFα cohort (age 30-50 years), before and after propensity score matching

	Autoimmune disorders cohorts (age 30-50 years)[a]			
	Before matching		After matching	
	No-TNFα N=25,000[b]	Yes-TNFα N=4,397	No-TNFα N=4,340	Yes-TNFα N=4,340
**Duration of follow-up, mean [SD] days**	1477.4 [1015.2]	1611.6 [1078.0]	1348.5 [976.1]	1618.1 [1078.6]
**Sex, n (%) female**	15837 (63.3%)	2987 (65.9%)	2864 (66.0%)	2850 (65.7%)
**Age, years**				
Mean [SD]	40.6 [6.0]	40.8 [6.1]	40.9 [5.9]	40.8 [6.1]
Distribution, n (%)				
30-39	11284 (45.1%)	1869 (42.5%)	1906 (43.9%)	1860 (42.9%)
40-50	13716 (54.8%)	2528 (57.5%)	2434 (56.1%)	2480 (57.1%)
**Race/ethnicity, n (%)**				
White	14436 (57.7%)	2791 (63.3%)	2734 (63.0%)	2748 (63.3%)
Black	2146 (8.6%)	233 (5.3%)	268 (6.2%)	32 (5.3%)
Asian	319 (1.3%)	49 (1.1%)	61 (1.4%)	49 (1.1%)
Other[c]	8099 (32.4%)	1324 (30.1%)	1277 (29.4%)	1311 (30.2%)
**CCI**				
Mean score [SD]	0.6 [1.0]	0.6 [0.9]	0.7 [1.1]	0.6 [0.9]
Component disorders, n (%)				
Malignancy	349 (1.4%)	32 (0.7%)	60 (1.4%)	31 (0.7%)
Metastatic solid tumor	167(0.8%)	1 (<0.1%)	3 (0.1%)	1 (<0.1%)
Diabetes	2206 (8.8%)	306 (7.0%)	401 (9.2%)	300 (7.0%)
Diabetes w/ complications	395 (1.5%)	51 (1.2%)	78 (1.8%)	50 (1.2%)
Congestive heart failure	223 (1.0%)	19 (0.4%)	50 (1.2%)	17 (0.4%)
Myocardial infarction	76 (0.3%)	6 (0.1%)	11 (0.3%)	6 (0.1%)
Peripheral vascular disease	293 (1.2%)	19 (0.4%)	41 (0.9%)	19 (0.4%)
Chronic pulmonary disease	3800 (15.2%)	408 (9.3%)	669 (15.4%)	402 (9.3%)
Cerebrovascular disease	319 (1.3%)	30 (0.7%)	52 (1.2%)	29 (0.7%)
Dementia	13 (<0.1%)	1 (<0.1%)	1 (<0.1%)	1 (<0.1%)
Hemiparaplegia	69 (0.3%)	9 (0.2%)	9 (0.2%)	9 (0.2%)
Mild liver disease	666 (2.7%)	86 (2.0%)	114 (2.6%)	86 (2.0%)
Severe liver disease	36 (0.2%)	3 (0.1%)	7 (0.2%)	3 (0.1%)
Renal disease	313 (1.3%)	33 (0.8%)	56 (1.3%)	33(0.8%)
Peptic ulcer disease	205 (0.8%)	36 (0.8%)	35 (0.8%)	35 (0.8%)
Rheumatic disease	4523 (18.1%)	1612 (36.7%)	963 (22.2%)	1575 (36.3%)
HIV	85 (0.3%)	1 (<0.1%)	22 (0.5%)	1 (<0.1%)

Caption: Grey highlight indicates characteristics with standardized mean difference >0.25 between groups. Patients in the two cohorts were propensity score-matched on sex, age, race/ethnicity, and CCI score. Abbreviations: CCI, Charlson Comorbidity Index; EHR, electronic health records; HIV, human immunodeficiency virus; SD, standard deviation; anti-TNFα, tumor necrosis factor-alpha inhibitor. Notes: [a] Demographic and clinical characteristics are among patients who met the inclusion criteria for history (90 days) and follow-up (6 months) in the EHR database. [b] The cohort was comprised of 25,000 randomly selected patients with no ant-TNFα use. [c] Other' includes all other race/ethnicities as well as when this information was missing.

Table S6. Demographic and clinical characteristics of the Yes-TNFα cohort and a randomly selected No-TNFα cohort (age 51-70 years), before and after propensity score matching

	Autoimmune disorders cohorts (age 51-70 years)[a]			
	Before matching		After matching	
	No-TNFα N=25,000[b]	Yes-TNFα N=6,868	No-TNFα N=6,412	Yes-TNFα N=6,412
**Duration of follow-up, mean [SD] days**	1454.3 [1003.6]	1586.2 [1023.7]	1453.3 [989.3]	1608.8 [1033.5]
**Sex, n (%) female**	16079 (64.3%)	4572 (66.6%)	4199 (65.5%)	4223 (65.9%)
**Age, years**				
Mean [SD]	60.2 [5.6]	59.1 [5.6]	59.2 [5.5]	59.1 [5.6]
Distribution, n (%)				
51-59	12226 (48.9%)	3877 (56.5%)	3660 (57.1%)	3581 (55.8%)
60-70	12774 (51.1%)	2991 (43.5%)	2752 (42.9%)	2831 (44.1%)
**Race/ethnicity, n (%)**				
White	15445 (61.8%)	4223 (65.9%)	4021 (66.8%)	3931 (65.3%)
Black	1986 (7.9%)	442 (6.9%)	434 (7.2%)	413 (6.9%)
Asian	182 (0.7%)	45 (0.7%)	54 (0.9%)	45 (0.7%)
Other[c]	7387 (29.5%)	1698 (26.5%)	1511 (25.1%)	1631 (27.1%)
**CCI**				
Mean score [SD]	2.8 [1.7]	2.5 [1.3]	2.6 [1.5]	2.5 [1.3]
Component disorders, n (%)				
Malignancy	1135 (4.5%)	147 (2.1%)	230 (3.6%)	144 (2.3%)
Metastatic solid tumor	66 (0.3%)	6 (0.1%)	12 (0.2%)	6 (0.1%)
Diabetes	4900 (19.6%)	986 (14.4%)	1129 (17.6%)	908 (14.2%)
Diabetes w/ complications	1223 (4.9%)	203 (3.0%)	266 (4.2%)	189 (3.0%)
Congestive heart failure	851 (3.4%)	113 (1.7%)	172 (2.7%)	111 (1.7%)
Myocardial infarction	281 (1.1%)	50 (0.7%)	75 (1.2%)	45 (0.7%)
Peripheral vascular disease	1211 (4.8%)	151 (2.2%)	248 (3.9%)	145 (2.3%)
Chronic pulmonary disease	5023 (20.1%)	894 (13.0%)	1219 (19.0%)	831 (13.0%)
Cerebrovascular disease	1095 (4.9%)	157 (2.3%)	215 (3.4%)	153 (2.4%)
Dementia	59 (0.2%)	6 (0.1%)	11 (0.2%)	6 (0.1%)
Hemiparaplegia	121 (0.5%)	8 (0.1%)	28 (0.4%)	8 (0.1%)
Mild liver disease	1170 (4.7%)	200 (2.9%)	271 (4.2%)	193 (3.0%)
Severe liver disease	78 (0.3%)	8 (0.1%)	16 (0.3%)	8 (0.1%)
Renal disease	1130 (4.5%)	163 (2.4%)	239 (3.7%)	151 (2.4%)
Peptic ulcer disease	369 (1.5%)	58 (0.8%)	79 (1.2%)	55 (0.9%)
Rheumatic disease	8592 (34.4%)	3848 (56.0%)	2446 (38.2%)	3531 (55.1%)
HIV	53 (0.2%)	6 (0.1%)	12 (0.2%)	5 (0.1%)

Caption: Grey highlight indicates characteristics with standardized mean difference >0.25 between groups. Patients in the two cohorts were propensity score-matched on sex, age, race/ethnicity, and CCI score. Abbreviations: CCI, Charlson Comorbidity Index; EHR, electronic health records; HIV, human immunodeficiency virus; SD, standard deviation; anti-TNFα, tumor necrosis factor-alpha inhibitor. Notes: [a] Demographic and clinical characteristics are among patients who met the inclusion criteria for history (90 days) and follow-up (6 months) in the EHR database. [b] The cohort was comprised of 25,000 randomly selected patients with no ant-TNFα use. [c] Other' includes all other race/ethnicities as well as when this information was missing.

Table S7. Demographic and clinical characteristics of the Yes-TNFα cohort with ≥6 months of anti-TNFα use and a randomly selected No-TNFα cohort, before and after propensity score matching

	Autoimmune disorders cohorts[a]			
	Before matching		After matching	
	No-TNFα N=25,000[b]	Yes-TNFα (6 months) N=4,733	No-TNFα N=4,520	Yes-TNFα (6 months) N=45,20
**Duration of follow-up, mean [SD] days**	1,418.8 [985.1]	1,835.6 [1,013.8]	1,475.2 [978.5]	1,848.2 [1,018.2]
**Sex, n (%) female**	16,211 (64.8%)`	3,139 (66.3%)	2,978 (65.9%)	2,976 (65.8%)
**Age, years**				
Mean [SD]	56.2 [17.5]	51.8 [14.2]	52.3 [17.3]	51.9 [14.2]
Distribution, n (%)				
18-29	2566 (10.3%)	405 (8.6%)	604 (13.4%)	380 (8.4%)
30-39	2524 (10.1%)	608 (12.8%)	602 (13.3%)	572 (12.7%)
40-49	3269 (13.1%)	1004 (21.2%)	709 (15.7%)	942 (20.8%)
50-59	4998 (20%)	1292 (27.3%)	916 (20.3%)	1250 (27.7%)
60-69	5471 (21.9%)	965 (20.4%)	942 (20.8%)	930 (20.6%)
70-79	4339 (17.4%)	383 (8.1%)	550 (12.2%)	372 (8.2%)
80-89	1833 (7.3%)	76 (1.6%)	197 (4.4%)	74 (1.6) %
**Race/ethnicity, n (%)**				
White	15421 (61.7%)	3118 (65.9%)	2927 (64.8%)	2958 (65.4%)
Black	1953 (7.8%)	281 (5.9%)	297 (6.6%)	272 (6%)
Asian	204 (0.8%)	43 (0.9%)	44 (1%)	42 (0.9%)
Other[c]	7422 (29.7%)	1291 (27.3%)	1252 (27.7%)	1248 (27.6%)
**CCI**				
Mean score [SD]	2.6 [2.4]	1.9 [1.7]	2.1 [2.1]	1.9 [1.7]
Component disorders, n (%)				
Malignancy	1129 (4.5%)	84 (1.8%)	150 (3.3%)	83 (1.8%)
Metastatic solid tumor	58 (0.2%)	2 (0.04%)	4 (0.1%)	2 (<0.1%)
Diabetes	4086 (16.3%)	525 (11.1%)	562 (12.4%)	502 (11.1%)
Diabetes w/ complications	1093 (4.4%)	108 (2.3%)	130 (2.9%)	104 (2.3%)
Congestive heart failure	987 (4.0%)	54 (1.1%)	117 (2.6%)	53 (1.8%)
Myocardial infarction	271 (1.1%)	29 (0.6%)	38 (0.8%)	27 (0.6%)
Peripheral vascular disease	1465 (5.9%)	81 (1.7%)	185 (4.1%)	80 (1.8%)
Chronic pulmonary disease	4893 (19.6%)	543 (11.5%)	744 (16.5%)	521 (11.5%)
Cerebrovascular disease	1294 (5.2%)	100 (2.1%)	162 (3.6%)	94 (2.1%)
Dementia	192 (0.8%)	2 (0.04%)	12 (0.3%)	2 (<0.1%)
Hemiparaplegia	99 (0.4%)	7 (0.6%)	7 (0.2%)	7 (0.2%)
Mild liver disease	833 (3.33%)	90 (1.9%)	125 (2.8%)	90 (2.0%)

Severe liver disease	55 (0.22%)	6 (0.1%)	8 (0.2%)	6 (0.1%)
Renal disease	1406 (5.62%)	82 (1.7%)	158 (3.5%)	80 (1.8%)
Peptic ulcer disease	344 (1.38%)	48 (1.0%)	38 (0.8%)	47 (1.0%)
Rheumatic disease	7152 (28.61%)	2250 (47.5%)	1358 (30.0%)	2123 (47.0%)
HIV	49 (0.2%)	0 (0%)	8 (0.2%)	0 (0%)

Caption: Grey highlight indicates characteristics with standardized mean difference >0.25 between groups. Patients in the two cohorts were propensity score-matched on sex, age, race/ethnicity, and CCI score. Abbreviations: CCI, Charlson Comorbidity Index; EHR, electronic health records; HIV, human immunodeficiency virus; SD, standard deviation; anti-TNFα, tumor necrosis factor-alpha inhibitor. Notes: [a] Demographic and clinical characteristics are among patients who met the inclusion criteria for history (90 days) and follow-up (6 months) in the EHR database. [b] The cohort was comprised of 25,000 randomly selected patients with no ant-TNFα use. [c] Other' includes all other race/ethnicities as well as when this information was missing.

Table S8. Demographic and clinical characteristics of the Yes-TNFα cohort with ≥12 months of anti-TNFα use and a randomly selected No-TNFα cohort, before and after propensity score matching

	Autoimmune disorders cohorts[a]			
	Before matching		After matching	
	No-TNFα N=25,000[b]	Yes-TNFα (12 months) N=3,516	No-TNFα N=3,466	Yes-TNFα (12 months) N=3,466
**Duration of follow-up, mean [SD] days**	1,418.8 [985.1]	2,014.9 [995.8]	1,521.1 [975.4]	2016.3 [999.0]
**Sex, n (%) female**	16,211 (64.8%)	2,298 (65.4%)	2,294 (66.2%)	22,61 (65.2%)
**Age, years**				
Mean [SD]	56.2 [17.5]	51.6 [14.2]	51.6 [17.4]	51.7 [14.2]
Distribution, n (%)				
18-29	2566 (10.3%)	303 (8.6%)	494 (14.3%)	294 (8.5%)
30-39	2524 (10.1%)	466 (13.3%)	476 (13.7%)	457 (13.2%)
40-49	3269 (13.1%)	742 (21.1%)	574 (16.6%)	728 (21%)
50-59	4998 (20%)	960 (27.3%)	681 (19.6%)	954 (27.5%)
60-69	5471 (21.9%)	704 (20%)	688 (19.8%)	697 (20.1%)
70-79	4339 (17.4%)	284 (8.1%)	408 (11.8%)	279 (8%)
80-89	1833 (7.3%)	57 (1.6%)	145 (4.2%)	57 (1.6%)
**Race/ethnicity, n (%)**				
White	15421 (61.7%)	2275 (64.7%)	2244 (64.7)	2238 (64.6)
Black	1953 (7.8%)	196 (5.6%)	236 (6.8)	194 (5.6)
Asian	204 (0.8%)	31 (0.9%)	27 (0.8)	31 (0.9)
Other[c]	7422 (29.7%)	1014 (28.8%)	959 (27.7)	1003 (28.9)
**CCI**				
Mean score [SD]	2.6 [2.4]	1.9 [1.7]	2 [2.1]	1.9 [1.7]
Component disorders, n (%)				
Malignancy	1129 (4.5%)	63 (1.8%)	99 (2.9%)	62 (1.8%)
Metastatic solid tumor	58 (0.2%)	2 (0.1%)	7 (0.2%)	2 (0.1%)
Diabetes	4086 (16.3%)	381 (10.8%)	407 (11.7%)	374 (10.8%)
Diabetes w/ complications	1093 (4.3%)	68 (1.9%)	89 (2.6%)	68 (2.0%)
Congestive heart failure	987 (3.9%)	35 (1%)	92 (2.7%)	35 9 (1.0%)
Myocardial infarction	271 (1.9%)	21 (0.6%)	27 (0.8%)	21 (0.6%)
Peripheral vascular disease	1465 (5.9%)	59 (1.7%)	120 (3.5%)	59 (1.7%)
Chronic pulmonary disease	4893 (19.6%)	377 (10.7%)	536 (15.5%)	374 (10.8%)
Cerebrovascular disease	1294 (5.2%)	79 (2.3%)	117 (3.4%)	77 (2.2%)
Dementia	192 (0.8%)	1 (<0.1%)	14 (0.4%)	1 (<0.1%)
Hemiparaplegia	99 (0.4%)	5 (0.1%)	7 (0.2%)	5 (0.1%)
Mild liver disease	833 (3.3%)	60 (1.7%)	88 (2.5%)	60 (1.7%)

Severe liver disease	55 (0.2%)	3 (0.1%)	5 (0.1%)	3 (0.1%)
Renal disease	1406 (5.6%)	60 (1.7%)	120 (3.5%)	59 (1.7%)
Peptic ulcer disease	344 (1.4%)	38 (1.1%)	38 (1.1%)	37 (1.1%)
Rheumatic disease	7152 (28.6%)	1659 (47.2%)	1001 (28.9%)	1635 (47.2%)
HIV	49 (0.2%)	0 (0%)	5 (0.1%)	0 (0%)

Caption: Grey highlight indicates characteristics with standardized mean difference >0.25 between groups. Patients in the two cohorts were propensity score-matched on sex, age, race/ethnicity, and CCI score. Abbreviations: CCI, Charlson Comorbidity Index; EHR, electronic health records; HIV, human immunodeficiency virus; SD, standard deviation; anti-TNFα, tumor necrosis factor-alpha inhibitor. Notes: [a] Demographic and clinical characteristics are among patients who met the inclusion criteria for history (90 days) and follow-up (6 months) in the EHR database. [b] The cohort was comprised of 25,000 randomly selected patients with no ant-TNFα use. [c] Other' includes all other race/ethnicities as well as when this information was missing.

Table S9. Demographic and clinical characteristics of the Yes-TNFα cohort with RA and a randomly selected No-TNFα cohort with RA, before and after propensity score matching

	RA cohorts[a]			
	Before matching		After matching	
	No-TNFα N=25,000[b]	Yes-TNFα N=6,824	No-TNFα N=6,517	Yes-TNFα N=6,517
**Duration of follow-up, mean [SD] days**	1281.9 [946.9]	1519.7 [1003.6]	1340.1 [966.9]	1528.3 [1009.6]
**Sex, n (%) female**	18802 (75.2%)	5205 (76.3%)	5001 (76.7%)	4958 (76.1%)
**Age, years**				
Mean [SD]	63 [13.7]	57.2 [13.0]	57.2 [14.3]	57.6 [12.9]
Distribution, n (%)				
18-29	478 (1.9%)	217 (3.2%)	255 (3.9%)	198 (3%)
30-39	1147 (4.6%)	502 (7.4%)	581 (8.9%)	462 (7.1%)
40-49	2597 (10.4%)	1125 (16.5%)	1154 (17.7%)	995 (15.3%)
50-59	5316 (21.3%)	1980 (29%)	1659 (25.5%)	1902 (29.2%)
60-69	7009 (28%)	1877 (27.5%)	1599 (24.5%)	1848 (28.4%)
70-79	6057 (24.2%)	939 (13.8%)	985 (15.1%)	928 (14.2%)
80-89	2396 (9.6%)	184 (2.7%)	284 (4.4%)	184 (2.8%)
**Race/ethnicity, n (%)**				
White	14643 (58.6%)	4367 (64%)	4081 (62.6)	4144 (63.6%)
Black	2564 (10.3%)	574 (8.4%)	612 (9.4)	551 (8.5%)
Asian	189 (0.8%)	51 (0.7%)	59 (0.9)	50 (0.8%)
Other[c]	7604 (30.4%)	1832 (26.8%)	1765 (27.1)	1772 (27.2%)
**CCI**				
Mean score [SD]	3.9 [2.2]	2.9 [1.7]	2.9 [1.8]	3 [1.7]
Component disorders, n (%)				
Malignancy	1333 (5.33%)	135 (1.98%)	174 (2.7%)	134 (2.1%)
Metastatic solid tumor	79 (0.32%)	10 (0.15%)	5 (0.1%)	10 (0.2%)
Diabetes	4560 (18.24%)	889 (13.03%)	774 (11.9%)	865 (13.3%)
Diabetes w/ complications	1287 (5.15%)	188 (2.75%)	153 (2.4%)	185 (2.8%)
Congestive heart failure	1261 (5.04%)	117 (1.71%)	131 (2.0%)	115 (1.8%)
Myocardial infarction	310 (1.24%)	56 (0.82%)	42 (0.6%)	53 (0.8%)
Peripheral vascular disease	1547 (6.19%)	181 (2.65%)	201 (3.1%)	180 (2.8%)
Chronic pulmonary disease	4981 (19.92%)	898 (13.16%)	1003 (15.4%)	875 (13.4%)
Cerebrovascular disease	1423 (5.69%)	195 (2.86%)	176 (2.7%)	194 (3.0%)
Dementia	244 (0.98%)	11 (0.16%)	17 (0.3%)	11 (0.2%)
Hemiparaplegia	85 (0.34%)	11 (0.16%)	8 (0.1%)	11 (0.2%)
Mild liver disease	850 (3.4%)	159 (2.33%)	154 (2.4%)	158 (2.4%)
Severe liver disease	43 (0.17%)	5 (0.07%)	7 (0.1%)	5 (0.1%)

Renal disease	1792 (7.17%)	204 (2.99%)	203 (3.1%)	203 (3.1%)
Peptic ulcer disease	342 (1.37%)	49 (0.72%)	55 (0.8%)	48 (0.7%)
Rheumatic disease	25000 (100%)	6824 (100%)	6517 (100%)	6517 (100%)
HIV	43 (0.17%)	3 (0.04%)	4 (0.1%)	3 (0.1%)

Caption: Grey highlight indicates characteristics with standardized mean difference >0.25 between groups. Patients in the two cohorts were propensity score-matched on sex, age, race/ethnicity, and CCI score. Abbreviations: CCI, Charlson Comorbidity Index; EHR, electronic health records; HIV, human immunodeficiency virus; RA, rheumatoid arthritis; SD, standard deviation; anti-TNFα, tumor necrosis factor-alpha inhibitor. Notes: [a] Demographic and clinical characteristics are among patients who met the inclusion criteria for history (90 days) and follow-up (6 months) in the EHR database. [b] The cohort was comprised of 25,000 randomly selected patients with no ant-TNFα use. [c] Other' includes all other race/ethnicities as well as when this information was missing.

Figure S2. Unmatched, basic-matched, and propensity score-matched comparisons of tinnitus incidence between patients with autoimmune disorders, by use of TNFα-AB (a-c) or TNFα-FP (d-f) compared to no anti-TNFα therapy

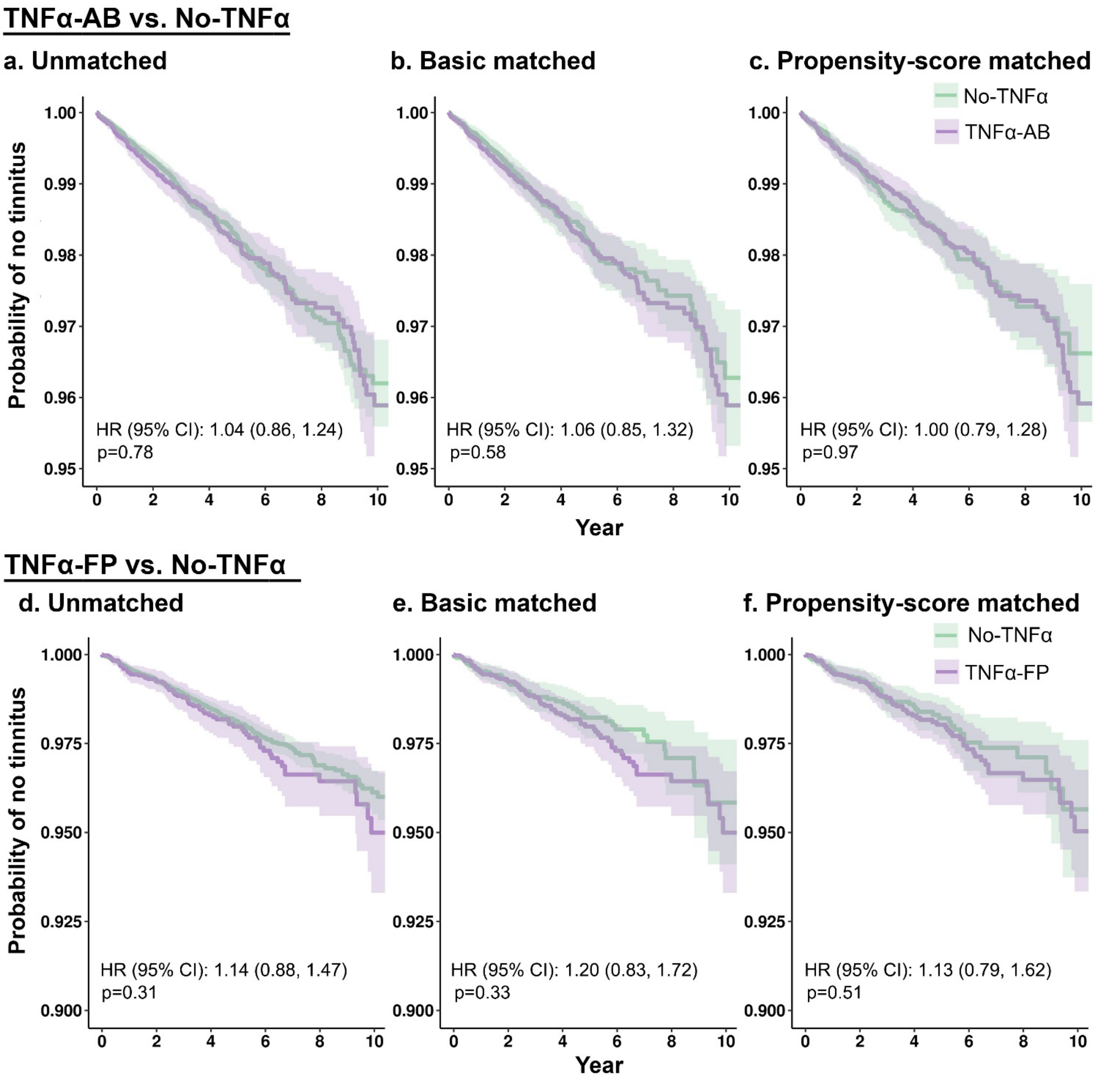

Caption: Abbreviations: AB, monoclonal antibody; anti-TNFα, tumor necrosis factor-alpha inhibitor; CI, confidence interval; FP, fusion protein; HR, hazard ratio.

Figure S3. Unmatched, basic-matched, and propensity score-matched comparisons of tinnitus incidence between patients with autoimmune disorders, by use of anti-TNFα for ≥6 months (a-c) or ≥12 months (d-f) compared to no anti-TNFα therapy

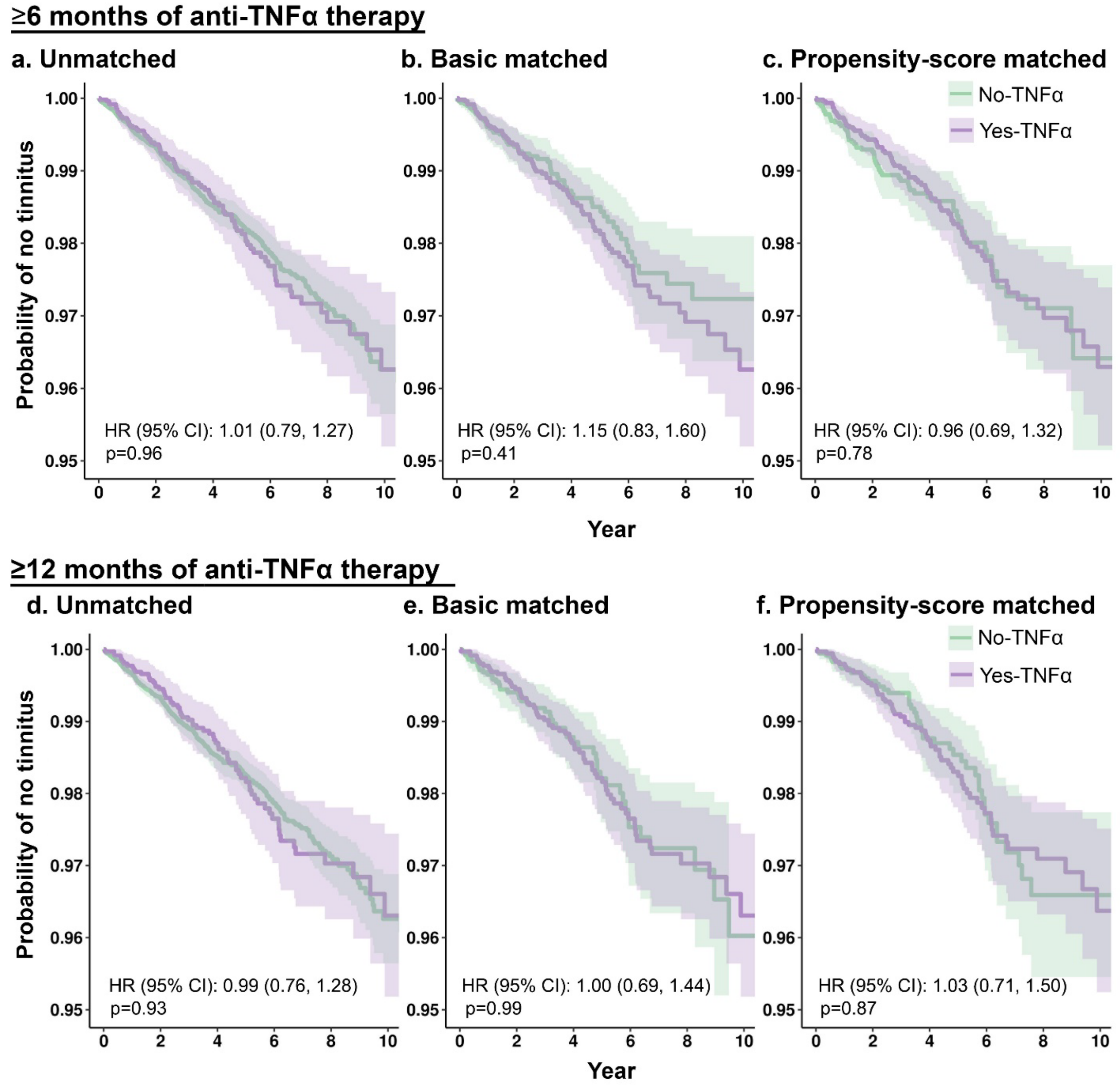

Caption: Abbreviations: anti-TNFα, tumor necrosis factor-alpha inhibitor; CI, confidence interval; HR, hazard ratio.

Figure S4. Unmatched (a), basic-matched (b), and propensity score-matched (c) comparisons of tinnitus incidence between patients with RA, by use of anti-TNFα therapy or no anti-TNFα therapy

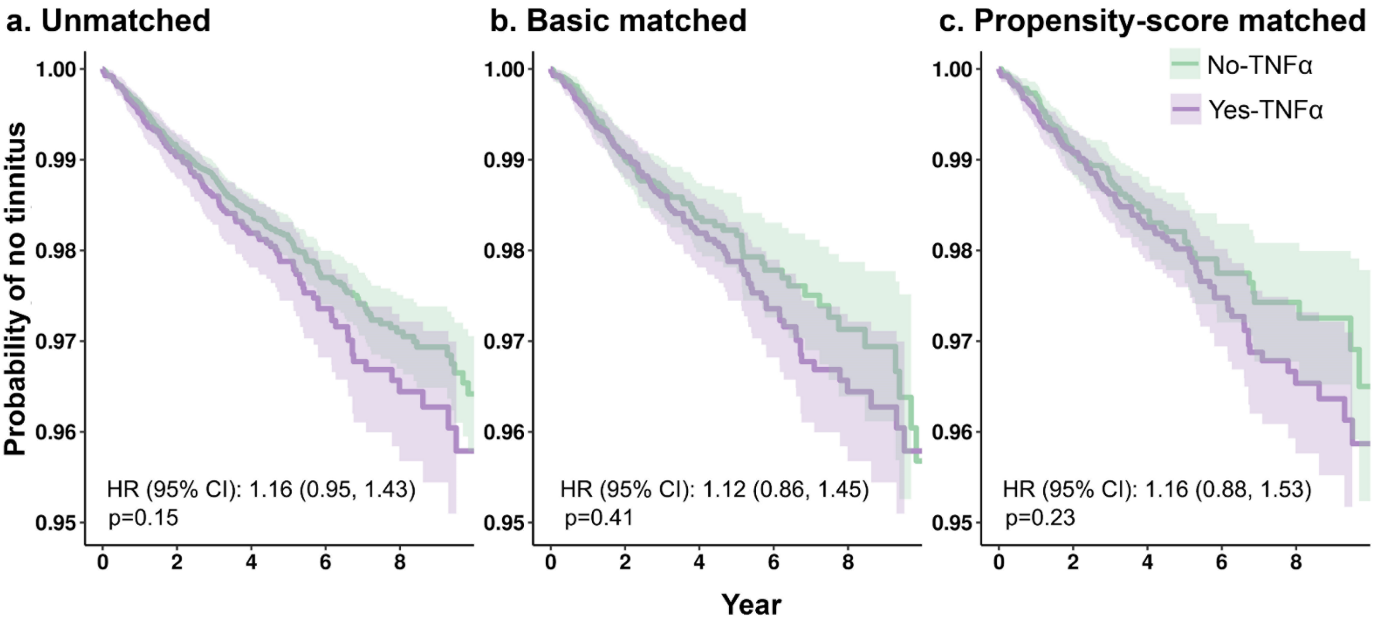

Caption: Abbreviations: anti-TNFα, tumor necrosis factor-alpha inhibitor; CI, confidence interval; HR, hazard ratio; RA, rheumatoid arthritis.
